# Supplementary material for: Hospital Admissions for Respiratory Tract Infections in Children Aged 0–5 Years for 2017/2023
Source: Front Pediatr. 2022 Jan 12;9:822985. doi: 10.3389/fped.2021.822985 (PMC8790534; doi:10.3389/fped.2021.822985)
Supplement: Supplementary file 1 [file Data_Sheet_1.PDF]

Supplementary file for the paper

Hospital admissions for respiratory tract infections in children aged 0-5 years for 2017/2023.

By Methi et al., 2021.

S-Table 1: ICD-10 codes and RTI categories. p. 2

S-Methods: Estimating projections. p. 3-4

S-Table 2: Observed vs. projected RTI-admissions + respiratory support. p. 5

S-Table 1: ICD-10 codes and RTI categories

Categories	ICD-10 codes
Upper RTI:	J00 **J02.0 J02.8** J02.9 **J03.0 J03.8** J03.9 J04.0 J04.1 J04.2 J05.0 J05.1 J06.0 J06.8 J06.9
Lower RTI:	**J12.0 J12.2 J12.3 J12.8** J12.9 **J13 J14 J15.0 J15.1 J15.2 J15.3 J15.4 J15.5 J15.6 J15.7 J15.8** J15.9 **J16.0 J16.8 17.0 J17.1 J17.2 J17.3 J17.8** J18.0 J18.1 J18.2 J18.8 J18.9 J22 **J20.0 J20.1 J20.2 J20.3 J20.4 J20.6 J20.7 J20.8** J20.9
Influenza-coded RTI:	**J09 J10.0 J10.1 J10.8** J11.0 J11.1 J11.8
RSV-coded RTI:	**J12.1 J20.5 J21.0**
COVID-19-coded RTI:	U07.1 U07.2
Respiratory support:	GXAV01 GXAV10 GXAV30

Note: Diseases with known pathogen in **bold**. Codes for respiratory support: Ventilation (GXAV01); Continuous Positive Airway Pressure (CPAP) (GXAV10); High flow oxygen therapy (GXAV30).

S-Methods: Estimating projections

Estimation:

First we estimate the number of monthly hospital admissions (y_t) using ordinary least square (OLS) regression. The regression model was set up as simple as possible to capture the seasonal variation, i.e. the average hospital admission per calendar month for each calendar year (i.e. m_1 = 1 if January if in January otherwise zero, m_2 = 1 if in February otherwise zero, ..., m_{11} = 1 if in November otherwise zero (December used as reference)), and a possible trend in hospital admissions over time (k_t, where $k_1 = 1$ in January 2017, $k_2 = 2$ in February 2017, ..., $k_{36} = 36$ in December 2019 and so on). This is illustrated in the following equation:

$$y_t = \beta_0 + \beta_1 * k_t + \beta_2 * m_1 + \beta_3 * m_2 + ... + \beta_{12} * m_{11} + \epsilon_t$$

From Figure 3 we see that the hospital admissions for the calendar months were very similar in the years 2017 through 2019, and thus this very simple model provides a rough but reasonable representation of the observed seasonal patterns. For three of the four scenarios ("business as usual", "children's immunity debt", and "maternal and child immunity debt") we run the regressions on the observations from January 2017 (t = 1) to December 2019 (t = 36), and used the estimated model — with the amendments described below — to predict the hospital admissions from August 2021 (t = 56) to June 2023 (t = 78) (see Figure 3). For "continuous lockdown" we run the regression on the observations from July 2020 (t = 43) to June 2021 (t = 54), and used the estimated model to predict the hospital admissions from August 2021 (t = 56) to June 2023 (t = 78) (see Figure 3).

Scenario projections:

To get the projected values we used the estimated models to predict admissions for months August 2021 (t = 56) to June 2023 (t = 78) using the following adjustments to the estimated model:

- "Business as usual": No adjustments.
- "Continuous lockdown": No adjustments.
- "Children's immunity debt": On top of the predictions from the estimated model, we added the difference between the estimated and observed number of hospital admissions for the period from January 2020 (t = 37) through July 2021 (t = 55), and divided the sum on each calendar month's average share of hospital admissions per season. For example, in January 2021 the predicted number of admissions was 932 and the observed was 132, yielding a difference $d_{Jan21} = 932 - 131 = 800$. The sum over all these differences (d = d_{Jan20} + d_{Feb20} + ... + d_{Jul21}) can be considered a simple estimate of the admissions that did not occur ("admission debt") because of the lockdown, and d = 5024. This admission debt was

then distributed to the 2021/22 season by the average share of all admissions in the seasons 2017-2019 that occurred in each calendar month (i.e. the shares that sum 100% were: 17% for January, 16% for February, 12% for March, 7% for April, 6% for May, 5% for June, 2% for July, 3% for August, 6% for September, 7% for October, 8% for November, and 11% for December).

- "Maternal and child immunity debt": We followed the same procedure as in the scenario "Children's immunity debt", except that we did it separately for children aged 0-12 months and 1-5 years. Moreover, the "admission debt" for the children aged 0-12 months ($d_{0-12months} = 2660$) was doubled (i.e. set to $d_{0-12months} = 2 * 2660 = 5320$) before added together with the "admission debt" for children aged 1-5 years ($d_{1-5years} = 2364$) and distributed across the calendar months of the season 2021/2022.

The exact same procedure was used for calculating the projected numbers of hospital admissions with respiratory support. All observed and projected numbers are given in S-Table 2.

S-Table 2: Observed vs. projected RTI-admissions + respiratory support.

	Hospital admissions					Respiratory support				
Month	Observed	Projected				Observed	Projected			
		BAU	CL	CID	MACID		BAU	CL	CID	MACID
Jan-17	1144					214				
Feb-17	933					158				
Mar-17	669					90				
Apr-17	427					31				
May-17	410					27				
Jun-17	346					19				
Jul-17	200					17				
Aug-17	213					18				
Sep-17	402					21				
Oct-17	476					28				
Nov-17	567					46				
Dec-17	600					63				
Jan-18	766					95				
Feb-18	838					129				
Mar-18	807					136				
Apr-18	531					78				
May-18	398					50				
Jun-18	344					31				
Jul-18	195					20				
Aug-18	275					21				
Sep-18	380					31				
Oct-18	416					33				
Nov-18	488					58				
Dec-18	744					111				
Jan-19	1027					209				
Feb-19	992					211				
Mar-19	661					124				
Apr-19	385					52				
May-19	368					36				
Jun-19	280					22				
Jul-19	190					14				
Aug-19	208					13				
Sep-19	374					35				
Oct-19	429					34				
Nov-19	486					44				
Dec-19	609					83				
Jan-20	640					89				
Feb-20	680					121				
Mar-20	451					76				
Apr-20	66					2				
May-20	77					6				
Jun-20	149					13				
Jul-20	133					5				
Aug-20	193					19				
Sep-20	236					14				
Oct-20	221					13				
Nov-20	218					16				
Dec-20	199					8				
Jan-21	132					2				
Feb-21	151					6				
Mar-21	187					9				
Apr-21	153					5				
May-21	192					10				
Jun-21	193					9				
Jul-21	159					6				
Aug-21	223	35	258	350	509	12	35	23	78	122
Sep-21	626	47	301	640	883	62	47	18	104	154
Oct-21	1211	50	286	744	1045	174	50	17	110	169
Nov-21		67	283	883	1288		67	20	149	235
Dec-21		104	264	1143	1630		104	12	229	341
Jan-22		197	197	1763	2664		197	6	419	686
Feb-22		190	216	1651	2585		190	10	405	675
Mar-22		141	252	1249	1940		141	13	300	511
Apr-22		78	218	740	1133		78	9	166	277
May-22		62	257	633	914		62	14	132	204
Jun-22		48	258	501	719		48	13	103	162
Jul-22		41	264	132	132		41	14	41	41
Aug-22		42	324	169	169		42	28	42	42
Sep-22		53	367	322	322		53	23	53	53
Oct-22		56	352	377	377		56	22	56	56
Nov-22		74	349	451	451		74	25	74	74
Dec-22		110	330	588	588		110	17	110	110
Jan-23		203	263	900	900		203	11	203	203
Feb-23		196	282	842	842		196	15	196	196
Mar-23		147	318	634	634		147	18	147	147
Apr-23		84	284	369	369		84	14	84	84
May-23		68	323	313	313		68	19	68	68
Jun-23		54	324	245	245		54	18	54	54

Note: BAU = Business as usual; CL = Continuous lockdown; CID = Children's immunity debt; MACID = Maternal and child immunity debt.
